# Endothelial-cell-mediated mechanism of coronary microvascular dysfunction leading to heart failure with preserved ejection fraction

**DOI:** 10.1007/s10741-022-10224-y

**Published:** 2022-03-09

**Authors:** Yong Wang, Juan Zhang, Zhen Wang, Cheng Wang, Dufang Ma

**Affiliations:** 1 Department of Cardiology, Affiliated Hospital of Shandong University of Traditional Chinese Medicine, 16369 Jingshi Road, Jinan, Shandong China; 2 Shandong University of Traditional Chinese Medicine, 4655 University Road, Changqing District, Jinan, Shandong China; 3 Department of Traditional Chinese Medicine, Zaozhuang Municipal Hospital, 41 longtou Road, Zaozhuang, China

**Keywords:** Endothelial cell, Coronary microvascular dysfunction, Heart failure with preserved ejection fraction

## Abstract

Although the prevalence of heart failure with preserved ejection fraction (HFpEF) is growing worldwide, its complex pathophysiology has yet to be fully elucidated, and multiple hypotheses have all failed to produce a viable target for therapeutic action or provide effective treatment. Cardiac remodeling has long been considered an important mechanism of HFpEF. Strong evidence has been reported over the past years that coronary microvascular dysfunction (CMD), manifesting as structural and functional abnormalities of coronary microvasculature, also contributes to the evolution of HFpEF. However, the mechanisms of CMD are still not well understood and need to be studied further. Coronary microvascular endothelial cells (CMECs) are one of the most abundant cell types in the heart by number and active players in cardiac physiology and pathology. CMECs are not only important cellular mediators of cardiac vascularization but also play an important role in disease pathophysiology by participating in the inception and progression of cardiac remodeling. CMECs are also actively involved in the pathogenesis of CMD. Numerous studies have confirmed that CMD is closely related to cardiac remodeling. ECs may serve a critical function in mediating the connection between CMD and HFpEF. It follows that CMECs participate in the mechanism of CMD leading to HFpEF. In this review article, we focus on the role of CMD in the pathogenesis of HFpEF resulting from cardiac remodeling and highlight the subsequent complexity of the EC-mediated correlation between CMD and HFpEF.

## Coronary microvascular dysfunction and heart failure with preserved ejection fraction

Heart failure (HF) is a complex syndrome of structural and functional disorders associated with reduced oxygen delivery to tissues. HF significantly reduces the quality of life of the patient and has a high morbidity and mortality rate. Despite various significant pharmacological advances, the burden of HF has been gradually increasing [1]. HF can be classified as either left ventricular systolic or diastolic dysfunction and can also be called HF with reduced ejection fraction (HFrEF), mid-range ejection fraction (HFmrEF), or preserved EF (HFpEF) [2]. Although most presentations are still characterized by a reduction in EF, the phenotype of HFpEF is becoming increasingly common, mainly due to improvements in diagnostic techniques, enhanced clinical awareness, and the gradual extension of lifespan. HFpEF accounts for 50% of HF admissions and shares similar mortality and morbidity to HFrEF [3].

Although the prevalence of HFpEF is growing worldwide, its complex pathophysiology has yet to be fully elucidated, and multiple hypotheses have all failed to produce a viable target for therapeutic action or provide effective treatment. HFpEF is identified as a heterogeneous syndrome linked to multiple comorbidities, such as diabetes mellitus, obesity, and hypertension [4]. Diastolic dysfunction has long been known as HFpEF, which is simply considered a mere problem of myocardial stiffness, mostly due either to changes in contractile proteins of cardiac myocytes or the effect of fibrosis on relaxation. This paradigm has recently been shifted with the discovery of several clues about the role of coronary microcirculation in the development of HFpEF [5]. Coronary microcirculation has become a significant research area of interest given the complex pathophysiology of HFpEF.

Coronary microcirculation is a complex net of arterioles, venules, and capillaries that enables the exchange of nutrients and waste metabolites between the vasculature and myocardium [6]. The blood supply of cardiac tissue is guided by the metabolic demands of the tissue. The coronary microcirculatory system could be said to be highly adaptive, with fast mechanisms such as adjustment of blood flow by changes in basal vascular tone that maintain oxygen demand in cardiac tissue [7]. However, microcirculatory remodeling is seen under various kinds of pathologic conditions, such as hypertension, hypertrophic cardiomyopathy, and myocardial infarction [8].

In various cardiovascular diseases, coronary microvascular dysfunction (CMD) manifests as structural and functional abnormalities of the coronary microvasculature. The etiology of CMD is related to endothelial dysfunction, microvascular spasm/sympathetic dysfunction, primary impairment of smooth muscle cell relaxation, etc. Microvascular dysfunction typically results in increased heterogeneity of blood flow and oxygen, which causes local hypoxia in myocardial tissue, ultimately leading to the detection of contractile and diastolic abnormalities [9, 10]. CMD has been hypothesized to cause myocardial oxygen disbalance in the failing heart [11].

### Epidemiology

In a prospective observational study, Dryer et al. [12] first reported CMD in patients with HFpEF based on the invasively determined coronary flow reserve (CFR) and the index of microvascular resistance (IMR). The CFR was lower and the IMR was higher in HFpEF patients than in those without HFpEF. A total of 71.4% of patients without HFpEF had normal coronary function, whereas most (73.4%) HFpEF patients had either an abnormal CFR or IMR.

To date, PROMIS-HFpEF (*n* = 202) is the largest prospective multicenter, multinational study to demonstrate a high prevalence of CMD in HFpEF by evaluation of CFR in subjects known to have HFpEF. CFR was measured in the PROMIS-HFpEF study by adenosine stress transthoracic Doppler echocardiography. The results showed evidence of CMD (defined as CFR < 2.5) in 75% of the HFpEF patients [13].

An interesting study by Taqueti et al. [14] has confirmed the relationship among CMD, diastolic dysfunction, and future risk of HFpEF in symptomatic patients without overt coronary artery disease. Impaired CFR has been independently associated not only with diastolic dysfunction but also with adverse events, especially HFpEF hospitalization.

### Possible mechanisms

This novel paradigm, in which CMD is central to HFpEF evolution, has gained support over several years. HFpEF patients are generally older and, more often than not, female, with multiple cardiovascular and noncardiovascular comorbidities (obesity, diabetes, hypertension, metabolic syndrome, hypercholesteremia, atrial fibrillation, chronic obstructive pulmonary disease, and even chronic kidney disease) [15]. Systemic inflammation and endothelial dysfunction are important indicators of these comorbidities that are also significantly involved in HFpEF pathophysiology [16].

The most accepted hypothesis that has been put forward is that systemic inflammation is the major factor contributing to myocardial performance by inducing CMD [17]. Circulating levels of inflammatory markers, including high-sensitivity(hs)-C-reactive protein (CRP), TNF-α, interleukin (IL)-6, and IL-1, are higher in HFpEF than in HFrEF. Observational studies have confirmed that biomarkers of inflammation are the best predictors of HFpEF severity and prognosis [18, 19].

Evidence has been accumulating in support of the links between inflammation and CMD and between CMD and HFpEF. Tona et al. [20] found that IL-6 and TNF-α were the sole determinants of CFR < 2.5, which suggests coronary microvascular impairment. The PROMIS-HFpEF study further validated that a low CFR is related to the severity of HFpEF, as marked by increased NT-proBNP and impaired right ventricular free wall strain [13].

A high burden of comorbidities generally leads to a systemic inflammatory state that impairs microvascular endothelial cells and leads to CMD. CMD impairs the perivascular environment and activates complex molecular pathways that eventually result in cardiac structural and functional dysfunction [21, 22].

For example, as approximately 50% of HFpEF patients are obese, obesity is an identified risk factor for HFpEF patients [23]. Hass et al. [24] found that HFpEF patients with an increased body mass index (BMI), i.e., ≥ 35 kg/m^2^, are at increased risk of death or cardiovascular hospitalization, independent of other key prognostic variables. Obesity is not merely a prominent comorbidity for HFpEF but also an important pathogenic factor involved in HFpEF pathogenesis. In obese human individuals, endothelium-related changes in myocardial blood flow were positively correlated with elevated leptin and CRP [25]. This result suggests that obesity significantly affects coronary circulatory function, which may contribute to decreased maximal myocardial blood flow and diastolic dysfunction [26].

## Cardiac remodeling and HFpEF

Cardiac remodeling is a complex process in which pathologic stimuli alter cardiac structure, shape, and function. Cardiac remodeling is a remarkable pathogenic manifestation of many serious cardiovascular diseases that ultimately progress to heart failure. Cardiac remodeling is closely related to the prognosis of clinical heart failure and has become an important therapeutic target for heart failure [27].

The remodeling process is clinically characterized by an increase in the ventricular cavity size. This complex process involves cardiomyocyte growth and death, vascular rarefaction, fibrosis, inflammation, and electrophysiological remodeling [28]. Both cardiac hypertrophy and cardiac fibrosis are common pathophysiological accompaniments to heart failure that are associated with systolic and diastolic dysfunction, arrhythmogenesis, and adverse outcomes [29, 30]. Both cardiomyocyte hypertrophy and interstitial fibrosis cause cardiac diastolic dysfunction, which is the major cardiac functional deficit in HFpEF [31].

## Cardiac remodeling and coronary microvascular dysfunction

It has been documented in numerous studies that ventricular remodeling in HFpEF is closely related to CMD. Investigations performed to date suggest that risk factor conditions (obesity, diabetes, hypertension, estrogen loss, inactivity, etc.) induce a proinflammatory, prooxidative state, resulting in both CMD and cardiac remodeling [32].

A few studies have assessed the relationship between coronary microvascular dysfunction and ventricular remodeling, mostly in myocardial infarction (MI) patients. Kitabata et al. [33] studied the prognostic significance of the microvascular resistance index (MVRI) immediately after PPCI with ventricular remodeling. A pressure guide wire was used to measure the MVRI of 24 patients as the ratio of the mean distal pressure to the average peak flow velocity during maximal hyperemia. Cardiac magnetic resonance was performed at baseline and at the 8-month follow-up to determine the degree of ventricular remodeling. Logistic regression analysis revealed that MVRI was the strongest univariate predictor of ventricular remodeling, where ventricular remodeling was more frequent in patients with an MVRI > 2.96 mmHg cm^−1^ s.

Cheng et al. [34] assessed the CFR in the noninfarcted myocardium of AMI patients. Decreased CFR in the remote region compared to a normal group suggested the common occurrence of microvascular dysfunction in the remote myocardium. Furthermore, the left ventricular end diastolic volume was higher in AMI patients with CFR > 2.05 than in the normal group. The results showed a link between the CFR value and adverse ventricular remodeling following AMI.

Gulati et al. [35] performed a study in which 65 dilated cardiomyopathy (DCM) patients and 35 healthy control patients were enrolled. Myocardial blood flow (MBF) and myocardial perfusion reserve (MPR) measurements were performed on all the patients using cardiovascular magnetic resonance to examine the relationship among myocardial perfusion, cardiac function, and replacement fibrosis. The data demonstrated that the DCM patients exhibited a higher rest MBF, but lower stress MBF and MPR, than the healthy patients. The results of the study confirmed that DCM patients exhibit microvascular dysfunction, the severity of which is associated with the extent of left ventricular impairment.

**Fig. 1:**
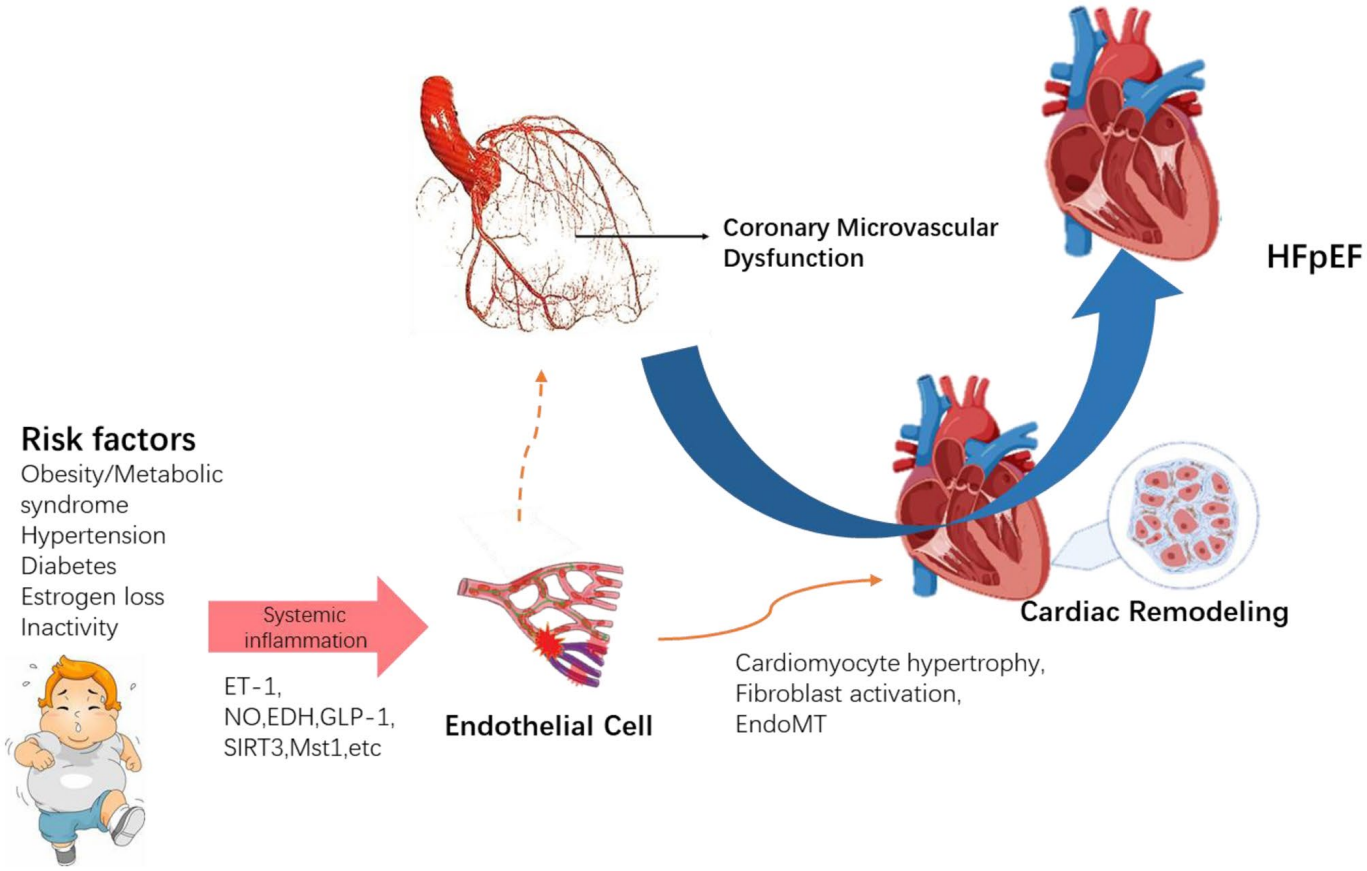
Possible contributing mechanisms to HFpEF development that converge at the level of CMD and endothelial cell dysfunction and ultimately lead to myocardial dysfunction

Despite strong evidence that CMD contributes to the evolution of HFpEF, the corresponding mechanisms are still not well understood and need to be studied further. Numerous studies have confirmed that CMD is closely related to cardiac remodeling. Cardiac remodeling, in turn, is considered to be the central player in HFpEF. Cardiac remodeling may serve a critical function in mediating the connection between CMD and HFpEF. ECs are not only important cellular mediators of cardiac vascularization but also play an important role in disease pathophysiology, participating in the inception and progression of cardiac remodeling. Endothelial cells are also actively involved in the pathogenesis of CMD. Thus, endothelial cells can be inferred to participate in the CMD mechanism leading to HFpEF (Fig. 1).

## Endothelial cell-mediated correlation between coronary microvascular dysfunction and HFpEF

### Endothelial cells and coronary microvascular dysfunction

Due to the high concealment and numerous etiologies of CMD, current understanding of the CMD pathophysiological mechanism is very limited. There appears to be a multiplicity of complex CMD mechanisms that frequently involve various combinations of several structural and functional changes in coronary microcirculation. Several potential mechanisms for CMD have been proposed, including enhanced coronary contractile response at the microvascular level (such as coronary microvascular spasm), damaged endothelium-dependent coronary vasodilator capacities, and increased coronary microcirculatory resistance due to structural changes (such as luminal narrowing, vascular remodeling, vascular rarefaction, and extramural compression) [36].

Structural or functional damage to coronary microvascular endothelial cells (CMECs) is a key mechanism in the occurrence and development of CMD [37]. Abnormalities in CMECs, which account for approximately 1/3 of the total number of cells in the heart, lead to impaired cardiac microvascular vessel integrity and subsequent CMD [38, 39]. The stimulation of pathological factors results in damage to several normal functions of CMECs, including proliferation, adhesion, migration, apoptosis, and secretion. Finally, this damage leads to abnormity of coronary microvascular systolic and diastolic function, increased coronary microcirculatory resistance, and decreased myocardial perfusion, all of which are early manifestations of CMD [39].

A bioptic study showed that activated endothelial cells (ECs) were a leading cause of microvascular spasm in patients with symptoms of angina pectoris or dyspnea and unobstructed coronary arteries [40]. Emerging evidence suggests ECs play specific roles in the pathogenesis of CMD. Endothelin-1 (ET-1), nitric oxide (NO), and endothelium-dependent hyperpolarization (EDH) factors are components of vasoactive substances secreted by vascular endothelial cells. These factors are considered to be biomarkers of vascular endothelial function. The ET-1 plasma concentration is the main systolic vascular factor and has been found to be noticeably increased in patients with coronary slow flow [41]. The main diastolic vascular factor, NO- and endothelium-dependent hyperpolarization-mediated digital vasodilatations, have been found to be markedly impaired in microvascular angina patients [42].

In addition to impairing systolic and diastolic function, ECs also cause CMD by deteriorating impaired cardiac microvascular vessel integrity. Microvascular structural changes play an important role in diabetic cardiovascular dysfunction. Endothelial apoptosis in diabetes may contribute to microvascular barrier dysfunction and rarefaction, which lead to CMD. GLP-1 can decrease the production of high glucose-induced reactive oxygen species and the apoptotic index, which, in turn, improves microvascular function [43]. The frequent occurrence of CMD is evidenced by a reduction in coronary flow reserve (CFR). He et al. [44] found that endothelial-specific SIRT3 KO (ECKO) mice exhibited myocardial capillary rarefaction together with a reduced CFR. In agreement with this result, an animal study by Lin et al. [45] showed that Mst1 inhibited autophagy and enhanced apoptosis in CMECs, thus participating in the pathogenesis of diabetic CMD.

### Endothelial cells and cardiac hypertrophy

ECs are not only important cellular mediators of cardiac vascularization but are also actively involved in the pathogenesis of heart failure. In fact, the interaction of ECs with their local environment modulates cardiac remodeling processes, and the balance of positive and negative signals determines the outcome of the remodeling response [46]. BRG1, a key component of the chromatin remodeling complex, is positively correlated with the pathogenesis of pathological cardiac hypertrophy in response to Ang II. Studies on mice constructed with endothelial-specific ablation of BRG1 have confirmed that BRG1 mediates Ang II-induced MRP8 production and macrophage homing to promote cardiac hypertrophy [47].

Cardiomyocytes play a key role in the pathogenesis of cardiac hypertrophy. Endothelial cells have been confirmed to communicate with adjacent cardiomyocytes, fibroblasts, and other cells resident in the heart by the release of growth factors, such as NO, ET-1, prostaglandins, or neuregulin-1 [48–51] (Table 1). The role of NO and ET-1 in cardiac hypertrophy has been intensively studied. Endothelial-cell-derived NO has been shown to have an inhibitory effect on cardiac hypertrophy. Liu et al. [48] reported that mice with endothelial-specific deletion of S1pr1 showed significantly aggravated cardiac dysfunction and deteriorated cardiac hypertrophy. The results of in vitro experiments have provided confirmation that S1P/S1pr1 activates the AKT/eNOS signaling pathway in ECs, leading to increased production of NO, an essential inhibitor of cardiomyocyte hypertrophy. Unlike NO, ET-1 promotes the formation of cardiac hypertrophy. Ang II stimulates ET-1 transcription, which, in turn, leads to cardiac hypertrophy [52]. Weng et al. [53] discovered that endothelial MRTF-A links ET-1 transactivation in ECs to cardiac hypertrophy via Ang II. In recent years, there has been considerably interest in neuregulin-1 (NRG-1), which belongs to the epidermal growth factor family and is expressed and released by CMECs. Vascular endothelial growth factor (VEGF) promotes NRG-1 release from ECs, and Akt, a signaling kinase that promotes myocyte growth, can be activated by NRG-1. VEGF is thought to contribute to cardiomyocyte hypertrophy by promoting the secretion of NRG-1 in ECs, because incubation of cardiomyocytes with conditioned medium from VEGF-treated ECs was found to result in increased phosphorylation of Akt [54].

**Table 1:** Summary of roles of EC-derived multifunctional cytokines in cardiac remodeling

Cytokines	Cardiomyocyte hypertrophy	Fibroblast activation	EndoMT	Reference
NO	−	−		[68, 69]
ET-1	+	+		[50, 65]
CTGF	−	+		[70, 71]
TGF-β1	+	+	+	[72–74]
NRG-1	+			[54]
Notch	−	+	+	[75–77]
HIF-α		+	+	[78, 79]

### Endothelial cells and cardiac fibrosis

Although fibroblasts are generally considered to be the central players in fibrosis, ECs are also important participants in the inception and progression of fibrosis. ECs can act as a source of myofibroblasts via the endothelial-mesenchymal transition (EndoMT) and therefore significantly contribute to the formation of extracellular matrix during fibrogenesis [55]. ECs also secrete numerous cytokines, which can positively or negatively affect cardiac remodeling [56] (Table 1).

EndoMT is considered to be the phenotypic transition of ECs into mesenchymal cells and has been increasingly receiving attention in recent years. During EndoMT, ECs lose some of their characteristics and acquire several characteristics of mesenchymal cells, such as loss of cell–cell adhesion, enhanced motility, and increased extracellular matrix protein secretion [57]. It has been suggested that a variety of stimuli, such as inflammation, growth factors, and hypoxia, regulate EndoMT via various signaling pathways and intracellular transcription factors.

The signaling cascade induced by the transforming growth factor-β (TGF-β) has been implicated as a central pathway in EndoMT [55]. This cascade facilitates the progression of cardiac fibrosis by regulating numerous transcriptional regulators, such as the Snail family of zinc finger transcription factors (Snail, Slug, Twist), as well as Zeb transcription factors (Zeb1 and Zeb2) [58, 59]. Among these transcription factors, Snail, Slug, and Twist have been proven to promote the occurrence of EndoMT. In a study by Xu [60], myocardial fibrosis was found to be considerably reduced in mice with endothelial Ets-1 deletion, which was accompanied by reduced EndoMT with decreased Snail, Slug, Twist, and ZEB1 expression. To further confirm the role of Ets-1 in EndoMT in vitro, cardiac endothelial cells were treated with TGF-β1, an inducer of EndoMT. In agreement with the in vivo findings, the expression levels of Snail, Slug, and Twist were decreased in Ets-1 knockdown cells.

The other key signal in cardiac fibrosis is the Notch pathway. This pathway mainly mediates the formation of myocardial fibrosis by promoting the expression of the Snail, Slug, and Zeb1 transcription factors during the EndoMT process. Frías et al. [61] found that active Notch expression promotes EndMT of aortic ECs, which results in upregulation of mesenchymal genes, such as those for fibronectin and Snail. Moreover, TGF-β1 exacerbates the Notch effect by increasing Snail1 and fibronectin activation.

Cross-talk between hypoxia and other signaling pathways has been shown to be a powerful inducer of EndoMT-related cardiac fibrosis. Hypoxia-inducible factor-1α (HIF-1α) has been recently recognized as an important molecule for the regulation of the EndoMT process under hypoxic conditions. HIF-1α can promote the EndoMT of human coronary endothelial cells by activating the transcription factor Snail [62]. Liu et al. [63] cultured human cardiac microvascular endothelial cells (HCMECs) in a strictly controlled hypoxic environment (1% O_2_). The results confirmed that hypoxia induces EndoMT in HCMECs mainly by activating TGF-β and Notch signaling.

In this section, we discuss EC-derived cytokines with known effects on cardiac fibrosis (Table 1). These ECs produce paracrine factors that can modulate fibroblast activation, fibroblast proliferation, and ECM deposition. These paracrine factors include NO, CTGF, TGF-β1, and ET-1 [56]. TGF-β1 is the most prominent cytokine implicated in myofibroblast transformation and fibrotic remodeling. Klf2 overexpression has been found to reduce Ang-II-elevated TGF-β1 expression in ECs. Moreover, conditioned medium from ECs overexpressing KLF2 has been found to attenuate cardiac fibroblast migration and proliferation, with associated reduced expression of α-SMA, collagen type I, and collagen type III. ET-1, mainly produced by ECs upon cardiac injury, is sufficient to promote fibroblast proliferation, activation and collagen synthesis. Conversely, endothelial-specific ET-1 knockout was found to remarkably improve myocardial fibrosis and cardiac function [64]. Zhang et al. [65] confirmed that the deletion of E26 transformation-specific (ETS)-related gene (ERG) in ECs promoted the secretion of ET-1, which subsequently accelerated the proliferation, phenotypic transition, and collagen production of cardiac fibroblasts in a paracrine manner. NO has been shown to be an essential cardioprotective factor. NO can protect the heart from cardiac fibrosis during the pathological processes of chronic heart failure [66]. The results of in vitro experiments have demonstrated that S1PR1-expressing HUVECs produced and secreted a high quantity of NO into a cell-culture supernatant, thus reducing fibroblast proliferation, fibroblast-to-myofibroblast differentiation, and extracellular matrix production in a paracrine manner [48]. Connective tissue growth factor (CTGF) is currently recognized as a potent profibrotic factor. Lee et al. [67] confirmed that snail-overexpressing ECs noticeably stimulated the transdifferentiation of fibroblasts to myofibroblasts via the secretion of CTGF.

## Conclusion

Structural and functional alterations in coronary microcirculation potentially lead to HFpEF. Improvements in the understanding of the pathophysiology of HFpEF, a disease affecting half of the total-heart-failure population, has increased the body of evidence on the involvement of CMD in HFpEF. CMD is closely related to cardiac remodeling, which ultimately leads to HFpEF. Cardiac remodeling is characterized by cardiac hypertrophy and myocardial fibrosis. Furthermore, ECs contribute to both CMD and cardiac remodeling.

**Fig. 2:**
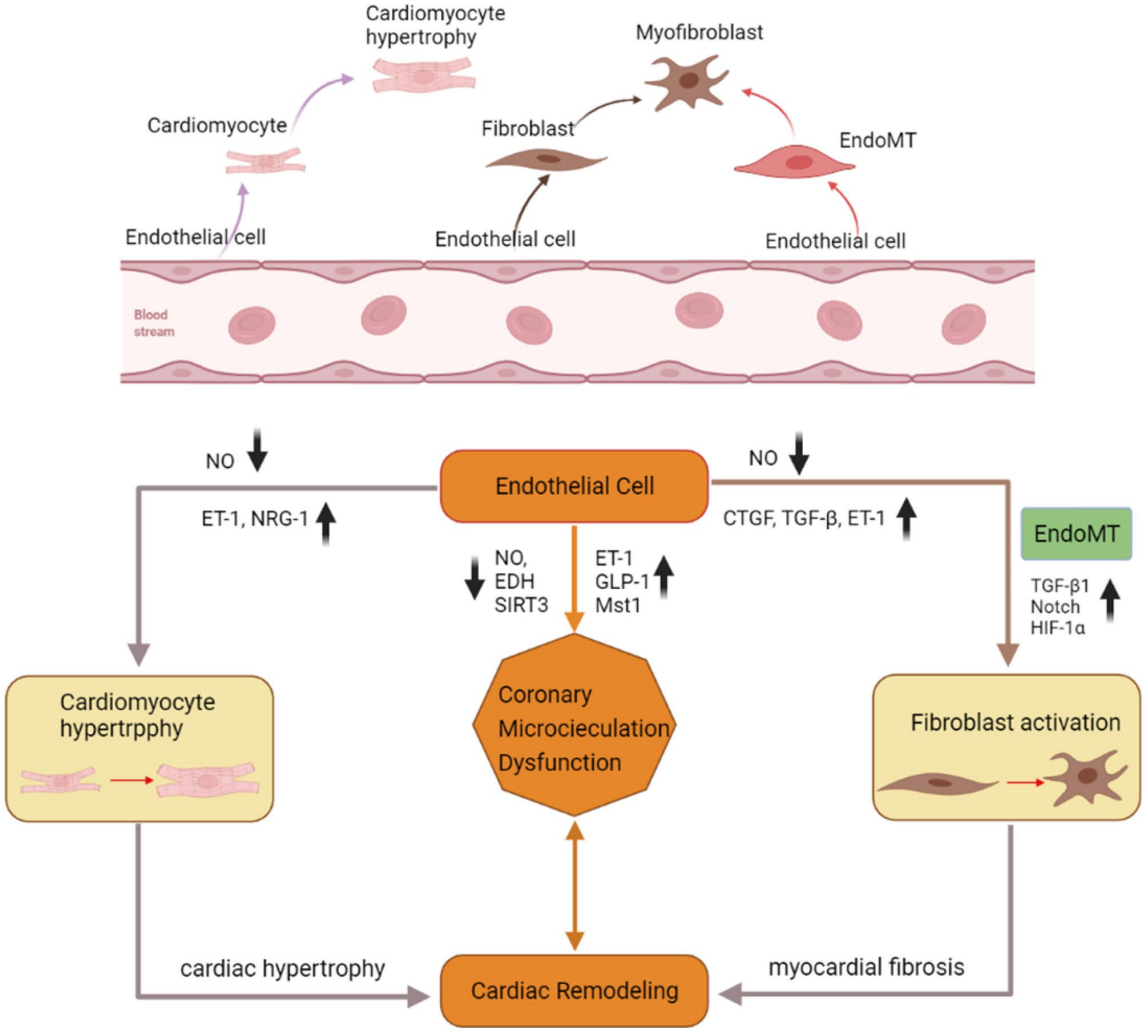
Impact of endothelial cell dysfunction on the pathogenesis of HFpEF. Comorbidities, such as obesity, hypertension, diabetes, estrogen loss, and inactivity, induce a systemic inflammatory state leading to endothelial cell dysfunction, and the resulting altered secretion levels of NO, EDH, ET-1, and GLP-1 promote CMD. The resulting CMD is closely related to cardiac remodeling. However, endothelial cell dysfunction contributes to cardiomyocyte hypertrophy and fibroblast activation by regulating the levels of a range of inflammatory cytokines, such as NO, ET-1, NRG-1, CTGF, and TGF-β, which eventually leads to cardiac remodeling. Furthermore, activation of the TGF-β-, Notch-, and HIF-1α-related pathways results in the endothelial-to-mesenchymal transition (EndMT), a process by which endothelial cells transdifferentiate into myofibroblasts, resulting in cardiac fibrosis

Structural or functional damage to coronary microvascular endothelial cells is the core process in the occurrence and development of CMD. ECs can contribute to cardiac hypertrophy and fibrosis via the endothelial-mesenchymal transition (EndoMT) or by secreting profibrotic and hypertrophic mediators. In this review, the importance of CMD in the pathogenesis of HFpEF resulting from cardiac remodeling is highlighted, and the subsequent complexity of the EC-mediated correlation between CMD and HFpEF is emphasized (Fig. 2).

HFpEF is a complex disorder caused by multifactorial stresses secondary to comorbidities. Only treatment of risk factors has been shown to be effective for preventing HFpEF thus far. Cardiac remodeling and CMD-modifying medications should be seriously considered during HFpEF treatment. EC damage is the key link between CMD and HFpEF. The next challenge is to find novel multidirectional strategies to abrogate endothelial dysfunction and subsequent cardiac remodeling and CMD. The numerous remaining gaps in our knowledge warrant further investigation of the association of ECs and HFpEF.
